# A Mobile App for Measuring Real Time Fatigue in Patients with Multiple Sclerosis: Introducing the Fimo Health App

**DOI:** 10.3390/brainsci11091235

**Published:** 2021-09-18

**Authors:** Jana Mäcken, Marie Wiegand, Mathias Müller, Alexander Krawinkel, Michael Linnebank

**Affiliations:** 1 Department of Health Economics and Clinical Epidemiology, University Hospital Cologne, 50935 Cologne, Germany; 2 Department of Psychology, University of Cologne, 50923 Cologne, Germany; marie.wiegand@outlook.de; 3 Fimo Health, 50827 Koln, Germany; mathias.muller@fimo.io (M.M.); Alexander.krawinkel@fimo.io (A.K.); 4 Evangelische Kliniken Gelsenkirchen, 45879 Gelsenkirchen, Germany; linnebank@evk-ge.de; 5 Faculty of Health, University Witten/Herdecke, 58455 Witten, Germany

**Keywords:** fatigue, multiple sclerosis, mHealth, intervention, mobile application

## Abstract

Although fatigue is one of the most disabling symptoms of MS, its pathogenesis is not well understood yet. This study aims to introduce a new holistic approach to measure fatigue and its influencing factors via a mobile app. Fatigue is measured with different patient-reported outcome measures (Visual Analog Scale, Fatigue Severity Scale) and tests (Symbol Digit Modalities Test). The influencing vital and environmental factors are captured with a smartwatch and phone sensors. Patients can track these factors within the app. To individually counteract their fatigue, a fatigue course, based on the current treatment guidelines, was implemented. The course implies knowledge about fatigue and MS, exercises, energy-conservation management, and cognitive behavioral therapy. Based on the Transtheoretical Model of Behavior Change, the design of the Fimo health app follows the ten strategies of the process of change, which is a proven approach to designing health intervention programs. By monitoring fatigue and individual influencing factors, patients can better understand and manage their fatigue. They can share their data and insights about fatigue and its influencing factors with their doctors. Thus, they can receive individualized therapies and drug plans.

## 1. Introduction

Worldwide, more than 2.3 million people suffer from multiple sclerosis (MS); thus, it is one of the world’s most common neurological diseases [1]. MS is a neurodegenerative disorder of the central nervous system affecting young and middle-aged people and is known to cause a variety of clinical symptoms such as neurological impairments, pain, and fatigue [2]. Fatigue can be defined as “a subjective lack of physical and/or mental energy that is perceived by the individual or caregiver to interfere with usual or desired activity” [3]. People living with MS (pwMS) describe fatigue as one of the most disabling symptoms, as it impacts daily living, leisure activities, and work, and reduces the quality of life [4]. Moreover, it is the main reason for early retirement among pwMS and, consequently, a burden for social security systems [5].

The pathogenesis of MS-related fatigue is not well understood yet [6]. Nevertheless, two types of fatigue can be distinguished: Primary fatigue arises directly from the disease mechanisms of MS, such as demyelination, inflammation, or axonal loss. Secondary fatigue, on the other hand, is the consequence of sleep problems, pain, medication, or deconditioning [7]. The National Multiple Sclerosis Society (2021) identified ten secondary causes that contribute to the severity of fatigue: stress, mood disorders such as depression or anxiety, poor diet, comorbidities, sleep disorders, nocturia, pain and spasticity, decreased physical activity/muscle weakness and deconditioning, environmental factors, and side effects from medication [8]. Due to its multifaceted origins and complexity, fatigue is difficult to treat [7]. Treatment recommendations suggest addressing secondary contributing factors first [8]. Although in some cases, disease-modifying drugs can be an effective solution for special cases of fatigue, fatigue is not clearly understood yet [9]. However, fatigue is highly individual, as these factors vary between patients. Meta-analyses support the benefit of non-pharmacological interventions, whereas the evidence regarding pharmacological treatments is not conclusive [10,11]. Energy-conservation management, exercising, and cognitive behavioral therapy were especially able to significantly reduce fatigue—up to 30% [12,13]. However, these interventions could be even more effective after taking the individual influencing factors of pwMS into account.

This study aims to introduce a new holistic approach to measure fatigue and the influencing factors via a mobile app. The objective of the Fimo health app is to help pwMS treat their fatigue. In the first step, fatigue and the influencing factors are measured. This information helps pwMS and their physicians to better understand the occurrence of fatigue and enable more tailored interventions. To individually counteract their fatigue, pwMS also have the opportunity to engage in a fatigue course. The course provides knowledge about fatigue and MS, exercises, energy-conservation management, and cognitive behavioral therapy.

The approach of the Fimo health app is based on the transtheoretical model of behavior change, which will be broadly introduced. The design of the app follows the ten strategies of the process of change, which is a proven approach in the design of health intervention programs. Each step and its implementation in the Fimo health app is explained. The discussion summarizes the current state of the art and its advantages for physicians.

## 2. Measuring Fatigue

Fatigue is, due to its multifaceted origins and complexity, difficult to capture [7]. However, two types of measures for fatigue can be distinguished: patient-reported outcome measures and measures of changes in motor or cognitive functions. Patient-reported outcome measures capture fatigue as a subjective symptom by addressing patients to rate their fatigue or different aspects of it. Performance-based measures, on the other hand, measure fatigue more objectively, e.g., based on the decline of cognitive processing speed [14]. Both types of measures are used to detect fatigue within the Fimo health app.

### 2.1. Patient-Reported Outcome Measures

**Figure 1:**
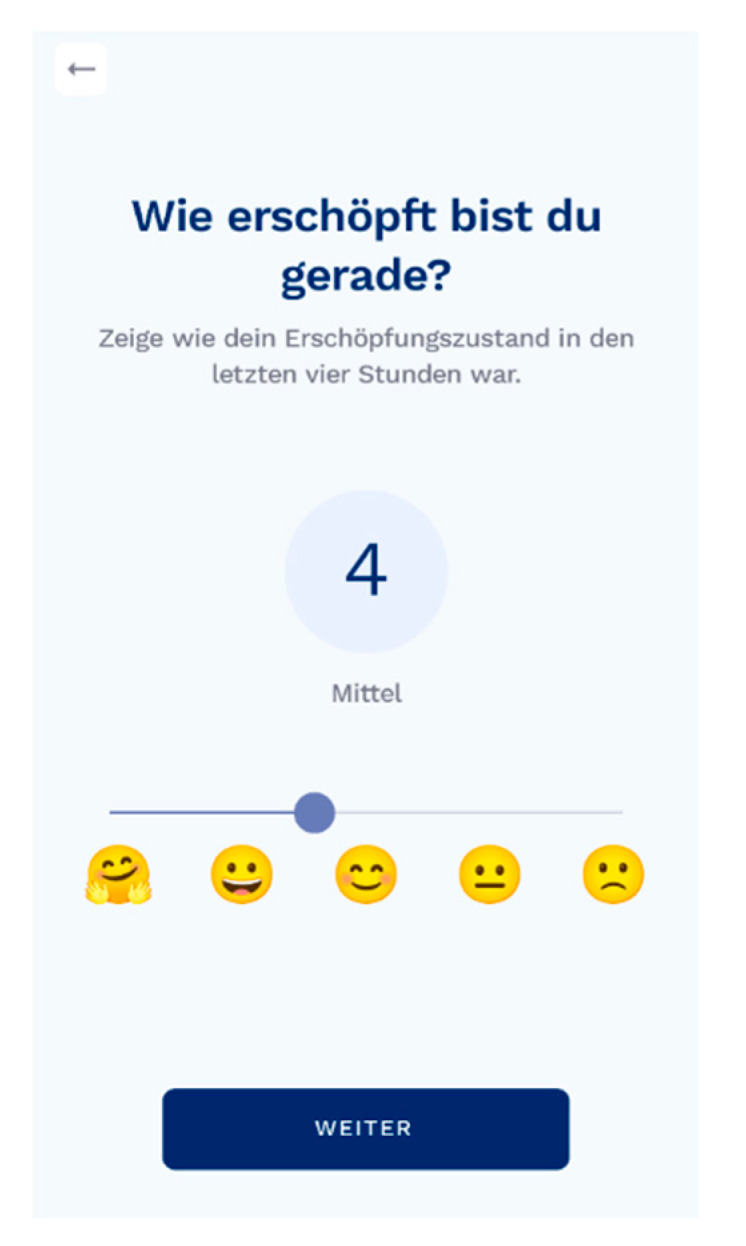
The VAS implemented in the Fimo health app.

Different patient-reported outcome measures are used to measure fatigue. The visual analog scale (VAS) is used to detect fatigue three times, or every four hours, during waking hours (Figure 1). PwMS have the option to set a reminder to register their VAS. PwMS are asked to rate their current level of fatigue on a scale from 0 to 10, whereby higher levels indicate more fatigue. Previous research showed that a very high correlation between VAS measurements and a series of drawings of faces with expressions of increasing distress [6,15]. Answering the VAS takes only a couple of seconds and has been shown to be a valid tool to measure fatigue among pwMS [16].

Besides the VAS, the Fatigue Severity Scale (FSS) is also used to detect fatigue [17]. The FSS is a nine-item instrument designed to assess fatigue as a symptom of several chronic conditions, including MS. PwMS answer the FSS once a week to detect long-term changes. The FSS has demonstrated good internal consistency, reliability, and validity [18]. Moreover, the Chalder Fatigue Scale is implemented, as it has shown to be more sensitive to change compared to the FSS [19]. The Chalder Fatigue Scale consists of eleven items covering physical and mental fatigue, and pwMS are asked to answer the questionnaire once a week. The completion of the Chalder Fatigue Scale only takes 2–3 min and has been validated among pwMS [20,21].

### 2.2. Performance-Based Measures for Fatigue

Fatigue correlates with declines in processing speed, reaction time, and/or accuracy over time, which are measured in different tests [22]. Performance-based tests capture the decline, thus measuring fatigue more objectively compared to self-reports. Among pwMS, the Symbol Digit Modalities Test (SDMT) is widely used to test cognitive declines in processing speed and has been proven to be valid and reliable [23]. During the 90 s assessment, pwMS are asked to sort nine numbers to different symbols according to the number. The score is the number of correct symbols. The SDMT was tested to be valid in a mobile and longitudinal setting with tests every three days [24]. Within the Fimo health app, pwMS are therefore asked to answer the test every three days.

### 2.3. Measuring Influencing Factors

Previous research identified several factors influencing fatigue, such as stress, mood disorders including depression or anxiety, poor diet, comorbidities, sleep disorders, nocturia, pain and spasticity, decreased physical activity or muscle weakness and deconditioning, environmental factors (temperature, humidity, light), and side effects from medication [8]. The vital parameters heart rate, stress level, steps, and sleep are measured with Garmin devices and are displayed within the Fimo health app.

The environmental factors temperature, humidity, light, and noise are captured by using the GPS location. Within the Fimo health app, users have the option to display the parameters and set them in relation to their individual fatigue level. All data are stored on German servers and anonymized in accordance with GDPR compliance.

## 3. Transtheoretical Model of Health Behavior Change

The transtheoretical model (TTM) of health behavior change is a dynamic model consisting of different stages of change and integrated processes and principles of interventions depending on individuals’ readiness to act on a new healthier behavior [25]. Six stages of health behavior change, based on people’s motivation, can be distinguished:

1. Precontemplation: people are not ready and do not intend to take action;
2. Contemplation: people are beginning to realize that their behavior is problematic;
3. Preparation: people are ready to take action in the immediate future;
4. Action: people take action and modify their health behaviors;
5. Maintenance: people sustain their actions over a longer period;
6. Termination: people do not have the temptation to switch back to old behaviors.

While moving through these stages, people apply different strategies and techniques, depending on their motivation and goals. These processes result in strategies that help people make and maintain change. These strategies and techniques are summarized as the ten processes of change, with some processes being more relevant to a specific stage of change than others [26]. These ten processes can be divided into inner cognitive-affective processes on the one hand and behavioral processes on the other hand. They are based on several major theories of intervention describing key ways in which people change their behaviors [27]. The TTM has been applied successfully in energy conservation and exercise programs for pwMS in previous research [28,29].

### Other Behavior Change Techniques

Alongside the TTM, we implemented various concrete health behavior change techniques extracted from the Taxonomy of Behavior Change Techniques by Abraham and Michie [30]. We combined the techniques to “provide information about behavior-health link”, (i.e., general information about behavioral risk—for example, susceptibility to poor health outcomes or mortality risk in relation to the behavior) with “prompt intention formation” (i.e., encouraging the person to decide to act or set a general goal). This combination has been shown to be particularly effective in mHealth apps for mental and physical health [31]. For prompting intention formation, pwMS are asked to formulate SMART goals. The SMART technique is used to ensure that goals are specific, measurable, attainable, relevant, and timely. Additionally, basic gamification elements—for instance, collecting points based on the participation in the fatigue course, rewards for daily usage, a point system for activity within the app, and weekly reports—are implemented.

## 4. Overview of the Fatigue Course

**Figure 2:**
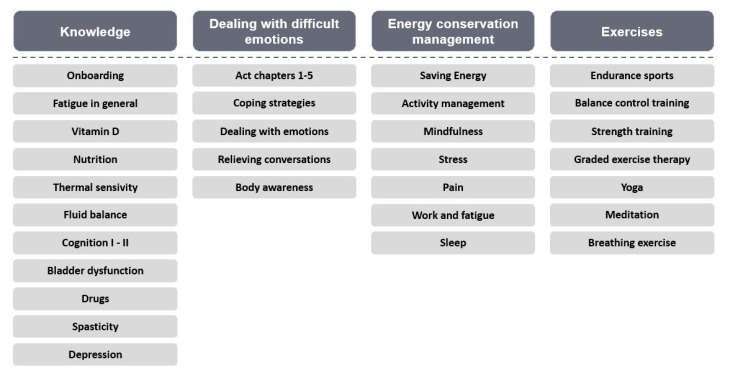
Overview of the Fimo fatigue course.

The Fimo fatigue course consists of eight weekly modules, which consist of individual daily chapters based on the current treatment guidelines [32]. The topics of the chapters can be divided into four main topics: basic knowledge about MS and fatigue, dealing with difficult emotions, exercises and energy-conservation management (Figure 2). Several studies and meta-analyses showed that these measures were most effective in treating fatigue among pwMS [10,33,34,35]. The selection of the topics is based on a systematic literature review on exercise and behavioral interventions for pwMS suffering from fatigue. Covering studies between 2006 and 2021, 467 studies were identified. After removing duplicates and excluding studies based on the exclusion criteria (no RCT, wrong type of intervention, non-MS sample, or no self-reported fatigue measure), 31 articles were included. Moreover, the design of the course was discussed through expert interviews with four neurologists. Additionally, a patient board was introduced to measure and improve the user experience of pwMS.

Different exercises are applied to treat fatigue and range from endurance sports, such as running, balance, strength, and aerobic exercises, to yoga. These exercises are explained in short videos, and pwMS can choose to complete the exercises that suit them most. The knowledge about MS and fatigue covers several relevant topics that are presented in an interactive way that includes dialogues, texts, graphics, and videos. The energy-conservation management section contains information and practical tips on how to structure daily routines to save energy. The content of the topic dealing with emotions and feelings is based on Acceptance and Commitment Therapy (ACT) [36], which is a psychotherapeutic approach that originated from Cognitive Behavioral Therapy (CBT). It combines classical behavior-therapy-oriented approaches with acceptance- and mindfulness-based techniques. Positive effects of ACT on fatigue among pwMS have been shown in previous research [37,38]. ACT techniques aim at psychological flexibility, which is described as being able to fully contact the present moment while acting in line with personal values [39]. The overarching goal is hence not a reduction of (physical) symptoms but an increase in the quality of life [40].

Specific ACT chapters were implemented in the fatigue course at several points. The four main topics concerning basic knowledge about MS and fatigue, how to deal with emotions and feelings, exercising with fatigue, and energy-conservation management are also to be found throughout the eight modules, repetitively.

### Ten Processes of Change within the Fatigue Course

The content of the Fimo fatigue course addresses the ten steps of the TTM. Meta-analyses showed greater effects in programs that are tailored to the ten steps of the transtheoretical model [41]. Thus, the fatigue course within the Fimo health app is designed on the ten processes of change to help pwMS to counteract their fatigue efficiently.

- Step 1: Consciousness raising:

The first step aims at increasing awareness about the causes and consequences of fatigue. This can be accomplished by providing information, education, or personal feedback concerning health behaviors. The fatigue course contains information about fatigue in general, as well as on interventions that have been proven to reduce fatigue, such as aerobic exercises, energy-conservation management, or meditation [11]. Furthermore, the vital and environmental factors are constantly monitored, and pwMS receive feedback on which factors influence their personal fatigue.

- Step 2: Dramatic relief:

In the second step, the experience of increased negative emotions, e.g., fear, anxiety, or worry, that goes along with one’s health problems, unhealthy behavior, and self-image, are at focus. It is followed by a reduced effect or anticipated relief if the appropriate action is taken [26]. However, the second step can also be positive if feelings such as inspiration or hope arise when hearing about a new solution or about how other people changed their health behaviors.

PwMS and fatigue particularly tend to struggle with catastrophizing and pressurizing thoughts [38], as well as worries about the future and negative feelings [42]. As these unpleasant inner experiences might be triggered in pwMS within the first chapter, they are provided with information on how to face these feelings in the chapter “dealing with emotions”. Practical exercises directly targeting these negative feelings, thoughts, and worries are provided in separate ACT chapters. They enable pwMS to distance themselves from their detrimental thoughts, shift their attention back into the present moment, and cope with difficult emotions. Hence, ACT is perfectly suitable to address the second step of the TTM.

- Step 3: Self-reevaluation:

This step is about realizing that health behavior change is an important part of one’s identity. It also involves appraising one’s values with respect to problematic behavior and creating a new self-image. Being aware and accepting one’s identity in all facets are core processes of ACT. Respective mindfulness exercises support the patients in letting go of their rigid self-image as MS patients and reevaluating their self-image. Moreover, pwMS learn about body-consciousness, how it might have changed due to their illness, and how to gain it back.

- Step 4: Environmental reevaluation:

Step four is about how the presence or absence of a personal behavior affects one’s social environment. In the case of fatigue, social relationships might suffer as pwMS are less available. Several chapters of the course address this aspect. PwMS are encouraged to practice gratitude while focusing on their social network in one of the ACT exercises. Moreover, they learn how they can address their social networks and share their sorrows in the chapter “relieving conversations”. Moreover, energy-conservation management methods and tips are taught to optimize time allocation and use times without fatigue more efficiently.

- Step 5: Self-liberation:

The self-liberation is both the belief that one can change and the commitment and re-commitment to act on that belief. It is about believing in one’s ability to change and making commitments to act on that belief. To encourage pwMS to commit to and increase exercising, a SMART-goal setting is applied at the beginning of the chapters that include exercises. Furthermore, possible barriers which could hinder reaching a goal are identified, and possible solutions are suggested. All methods and interventions applied in the Fimo fatigue course are based on evidence and the clinical guidelines on treating fatigue among pwMS [29]. The effectiveness of the interventions is summarized and shared with the users to encourage changing their health behavior.

As pwMS especially suffer from a reduced quality of life [39], the exercises of the remaining ACT chapters encourage pwMS to identify their personal values and implement these into their daily lives. Thus, they aim to improve their quality of life despite the physical changes that pwMS might have to go through. By developing concrete action plans, these ACT chapters further contribute to the fifth step of self-liberation and commitment.

- Step 6: Social liberation:

Step 6 is about noticing public support and realizing that society is supportive of healthy behaviors. PwMS learn how to address their networks as well as the workplace. More than 40% of pwMS reduce their numbers of working hours, and 25% retire earlier due to their fatigue [43]. By dealing openly with the illness at work, misunderstandings, such as complaints because of absenteeism, can be reduced, pwMS are likely to experience support from colleagues, and it is easier to accept the fact of a changed workability.

- Step 7: Counterconditioning:

In step 7, pwMS learn how they can substitute problematic behaviors with healthier behaviors. Several chapters of the course address this step. Within the last ACT chapter, pwMS learn how to identify possible barriers along their way towards healthier behavior as well as how to develop a step-by-step action plan. In the chapters concerning fatigue and daily routines, pwMS learn how to structure their daily routines effectively and improve their sleep and nutrition. Another important factor that has yet to be shown to be effective is stress management [44]. PwMS learn to implement breaks in their daily routines and do breathing exercises. Furthermore, they have the option to track their stress level in the Fimo health app and to analyze and reflect on the situations in which they experience stress.

- Step 8: Stimulus control:

This step is about removing cues for unhealthy habits and adding prompts for healthier alternatives. This step is addressed by providing the opportunity to track vital and environmental factors that influence fatigue. Thus, the possible causes of fatigue can be identified, and pwMS can modify their health behavior to tackle fatigue. PwMS can adjust their routines and implement, for example, mindfulness and/or exercises in their daily schedules, at whatever the best time is, based on their fatigue levels. Additionally, pwMS have the option to set reminders for exercising.

- Step 9: Contingency management:

Step 9 involves providing consequences for taking steps in a particular direction by rewarding continued healthy behavior. PwMS have the option to self-monitor their behavior based on the vital parameters measured and displayed in the app. Based on the activity measure, they can check if they have reached their exercising goal or, for example, if their stress levels decreased after applying mindfulness exercises over some time. Furthermore, gamification elements are implemented within the Fimo fatigue course, and pwMS can collect points based on their participation within the course as an additional motivation.

- Step 10: Helping relationships:

In this step, pwMS should find people that are supportive of their change and combine caring, trust, openness, and acceptance of the new behavior. Two modules in the Fimo fatigue course address this step: “relieving conversations” and “work and fatigue”. In “relieving conversations”, pwMS learn that they can and should accept help from friends and family. PwMS learn to handle feelings, such as being a burden to others because of their disease, and that they can better manage their disease with social support. The chapter “work and fatigue” further contributes to this step by encouraging PwMS to deal openly with the illness at work. Thus, hurdles and misunderstandings, such as complaints because of absenteeism, might be reduced. On the contrary, through open communication with their employer and colleagues, pwMS will probably even experience support in difficult situations.

## 5. Discussion

The aim of the Fimo health app was to offer a holistic approach for pwMS to tackle fatigue, which is described as one of the most disabling symptoms as it impacts daily living, leisure activities, work and reduces the overall quality of life [4]. Thus, fatigue and the influencing factors are measured within the Fimo health app. To individually counteract fatigue, an eight-week fatigue course based on the current treatment guidelines is implemented [32]. The fatigue course consists of four different pillars covering knowledge about MS and fatigue, dealing with difficult emotions, exercises, and energy-conservation management.

By doing so, the Fimo health app has several advantages for pwMS and physicians. Monitoring fatigue and the influencing factors helps pwMS to better understand and manage their fatigue as they know which factors matter. They can counteract by applying the learned methods according to their individual influencing factors. PwMS have the option to share their data and insights about fatigue and the influencing factors with their doctors, which we strongly advise. Thus, based on the new information, doctors can individualize therapies and drug plans, which would improve the treatment of pwMS substantially. Doctors would receive more objective information about the occurrence of fatigue and could measure the success of therapies. Compared to other solutions, the Fimo health app offers a more holistic approach by measuring fatigue and the influencing factors as well as offering a course.

However, the app also has some current limitations. At the moment, only Garmin devices can be connected. Integrating interfaces from other providers is intended for the future. Moreover, data can only be shared via a PDF export with doctors due to data protection reasons, which makes the implementation in the varied hospital information systems complex. Besides, we are working on a better interplay between our measured data and the fatigue course. In the long run, pwMS should receive recommendations about which exercise would help to reduce their fatigue based on their individual and current influencing factors. However, more data are needed to train the algorithm. The Fimo health app is not on the market yet, as we are in the process of applying for a Digitale Gesundheitsanwendung (DiGA).

Previous research showed that fatigue fluctuates over time and even throughout the day [45,46]. These fluctuations are difficult to capture. Mobile health solutions offer new possibilities, particularly for complex chronic diseases such as MS and fatigue. PwMS are especially suited to adopt mobile health solutions, as they usually show their first symptoms between the ages of 20 and 40 [47]. Hence, the Fimo health app is a useful tool to gain new insight into the occurrence and treatment of fatigue.
